# Roles of Cytochrome P4502E1 Gene Polymorphisms and the Risks of Alcoholic Liver Disease: A Meta-Analysis

**DOI:** 10.1371/journal.pone.0054188

**Published:** 2013-01-15

**Authors:** Tao Zeng, Fang-Fang Guo, Cui-Li Zhang, Fu-Yong Song, Xiu-Lan Zhao, Ke-Qin Xie

**Affiliations:** 1 Institute of Toxicology, School of Public Health, Shandong University, Shandong Province, Jinan City, People’s Republic of China; 2 Department of Pharmacy, Qilu Hospital of Shandong University, Shandong Province, Jinan City, People’s Republic of China; University of Navarra School of Medicine and Center for Applied Medical Research (CIMA), Spain

## Abstract

**Background:**

Previous studies investigating the association between cytochrome P4502E1 (*CYP2E1*) polymorphisms and the risk of alcoholic liver diseases (ALD) have yielded conflicting results. Thus, a meta-analysis was performed to clarify the association between *CYP2E1* polymorphisms and the risks of ALD.

**Methods:**

A comprehensive literature search was conducted to identify the relevant studies. The fixed or random effect model was selected based on the heterogeneity test among studies. Publication bias was estimated using Begg’s funnel plots and Egger’s regression test.

**Results:**

A total of 27 and 9 studies were finally included for the association between the *CYP2E1 Pst I/Rsa I* or *Dra I* polymorphisms and the risks of ALD, respectively. Overall, the combined results showed that homozygous genotype c2c2 was significantly associated with increase risk of ALD in worldwide populations (c2c2 *vs.* c1c1: OR = 3.12, 95%CI 1.91–5.11) when ALD patients were compared with alcoholics without ALD. Significant associations between *CYP2E1 Pst I/Rsa I* polymorphism and ALD risk were also observed in Asians (c2c2 *vs.* c1c1: OR = 4.11, 95%CI 2.32–7.29) and in Caucasians (c2c2/c1c2 *vs.* c1c1: OR = 1.58, 95%CI 1.04–2.42) when ALD patients were compared with alcoholics without ALD. However, subgroup analysis stratified by ALD types showed that *CYP2E1 Pst I/Rsa I* polymorphism was not significantly associated with the risks of alcoholic cirrhosis (ALC). No significant association was observed between *CYP2E1 Dra I* polymorphism and ALD risks.

**Conclusion:**

This meta-analysis suggested that *CYP2E1 Pst I/Rsa I* polymorphism might be not significantly associated with advanced form of ALD (ALC), but might be significantly associated with other form of ALD such as steatosis, hepatisis, fibrosis. Furthermore, *CYP2E1 Dra I* polymorphism might be not significantly associated with the ALD risks. Since potential confounders could not be ruled out completely, further studies were needed to confirm these results.

## Introduction

Alcoholic liver disease (ALD) remains to be one of the most common etiologies of liver diseases and is a major cause of morbidity and mortality worldwide [1]. The burden of ALD is highest in the developed world, where it may account for as much as 9.2% of all disability-adjusted life years [2]. Although the progression of ALD has been well-characterized, there is no universally accepted therapy available to halt or reverse this process in humans [3]. At present, the underlying mechanisms of ALD are still not fully understood, however, oxidative stress has been demonstrated to play important roles [4]. With better understanding of the mechanisms and risk factors that mediate the initiation and progression of ALD, rational targeted therapy can be developed to treat or prevent ALD. It has been demonstrated that a clear correlation exists between cumulative alcohol intake and ALD; however, only a small portion of the alcohol abusers develop signs of liver disease, which suggests some of the genetic variations are involved in the etiology of ALD [5].

Cytochrome P4502E1 (CYP2E1) is a member of the phage I detoxifying enzymes, which plays important roles in the metabolic activation of many xenobiotics including alcohol. CYP2E1 is physiologically responsible for about 10% of ethanol metabolism, but it could be induced after chronic ethanol administration [6], [7]. Accumulating evidence has demonstrated that CYP2E1 activation may play crucial roles in the etiology of ALD, which might be related with overproduction of reactive oxygen species (ROS) and the enhancement of the lipid peroxidation [1], [8], [9]. Therefore, it is plausible that functional polymorphisms in *CYP2E1* gene might be related with the risk of ALD in individuals.

*CYP2E1*, located in 10q2403-qter, is a 1104 kb gene consisting of 9 exons and 8 introns. *CYP2E1* contains six restriction fragment length polymorphisms (RFLP), among which the *Pst I/Rsa I* polymorphism in its 5′-flanking region was reported to be associated with higher transcription and increased enzyme activity [10]. Another polymorphism detectable with *Dra I* in intron 6 is also known to be associated with increased expression and enzyme activity [11]. The associations between the above two *CYP2E1* gene polymorphisms and the risks of ALD have been widely investigated in the past 20 years. Unfortunately, these epidemiological studies have yielded conflicting results, which might be related with the ethnic difference in the distribution of the mutant alleles and the relatively small sample size in some studies underpowered to detect the potential effects. Considering the important roles of CYP2E1 in the etiology of ALD, it would be necessary to summarize all these individual studies and discern whether *CYP2E1* gene polymorphisms are associated with the risks of ALD. Thus, we performed a meta-analysis in order to provide more accurate estimate of the association of the above gene polymorphisms and the risks of ALD.

## Materials and Methods

### Literature and Search Strategy

A computerized literature search was conducted for the relevant available studies from 3 databases including PubMed, ISI Web of Science, and Embase. The search strategy to identify all possible studies involved use of combinations of the following key words: (“cytochrome P450 2E1” or “CYP2E1”) and “polymorphism” and (“alcohol” or “ethanol”) and (“alcoholic liver disease” or “ALD” or “alcoholic fatty liver” or “steatosis” or “hepatitis” or “fibrosis” or “cirrhosis”). The reference lists of review articles, clinical trials, and meta-analyses were also hand-searched to identify additional works. There was no restriction on time period, sample size, population, language, or type of reports. If more than one article were published using the same case series, only the study with largest sample size was selected. The literature search was updated to June 2012.

### Inclusion Criteria

The studies included must meet the following criteria: (1) evaluating the association between *CYP2E1 Pst I/Rsa I and/or Dra I* polymorphisms and the risk of ALD; (2) providing sufficient data for calculation of odds ratio (OR) with the corresponding 95% confidence interval (95%CI). When genotype frequencies and OR with 95%CI were all not available, authors were contacted to request the relevant information. All identified studies were carefully reviewed independently by two investigators to determine whether an individual study was eligible for inclusion in this meta-analysis.

### Data Extraction

Data were extracted independently by two investigators who reached a consensus on all of the items. The following information was extracted from each study: (1) name of the first author; (2) year of publication; (3) country of origin; (4) ethnicity of the study population; (5) numbers of cases and controls; (6) gender and age of enrolled subjects; and (7) numbers of genotypes in cases and controls.

### Statistical Analysis

The associations between *CYP2E1 Pst I/Rsa I and/or Dra I* polymorphisms and ALD risks were estimated by calculating pooled ORs and 95%CI. The comparisons were made between ALD patients and alcoholics without ALD, and between ALD patients and non-alcoholics without liver diseases (non-alcoholics), respectively. The significance of the pooled effect size was determined by *Z* test. χ2 analysis with exact probability was used to test departure from Hardy-Weinberg equilibrium (HWE) for the genotype distribution in control groups (non-alcoholics). Heterogeneity among studies was assessed by χ^2^-based Q test as well as the *I^2^* statistic [12]. A significant Q-statistic (*P <* 0.10) indicated heterogeneity across studies. Subgroup analyses were performed based on the ethnicity of the enrolled subjects, the type of ALD, and the gender of subjects. Sensitivity analysis was undertaken by removing one individual study each time to check whether any of single study could bias the overall estimate [13]. An individual study was suspected of excessive influence, if the point estimate of its omitted analysis lies outside of the 95%CI of the combined analysis. Begg’s funnel plots and Egger’s regression test were undertaken to assess the potential publication bias. Probability less than 0.05 was judged significant except for the *I* ^2^ statistic. Data analysis was performed using STATA version 11 (StataCorp LP, College Station, Texas, USA).

## Results

### Characteristics of Studies

**Figure 1:**
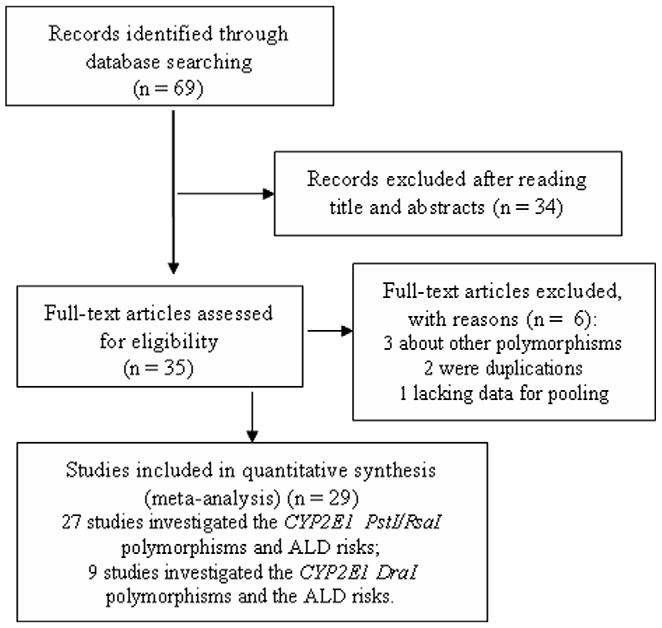
Flow chart of study selection based on the inclusion and exclusion criteria.

The flowchart of the study selection for this meta-analysis was shown in the **Figure 1**. As shown in **Figure 1**, a total of 69 studies were identified through database searching, and 40 studies were excluded for various reasons. Finally, 27 [5], [11], [14]–[38] and 9 studies [11], [17], [19], [23], [26], [31], [38]–[40] were included in the meta-analyses for the associations between the *CYP2E1 Pst I/Rsa I* polymorphism and the risks of ALD, and *CYP2E1 Dra I* polymorphism and the risks of ALD, respectively. All these included studies used peripheral blood samples for DNA extraction and polymerase chain reaction-restriction fragment length polymorphism (PCR-RELP) methods for genotyping. These studies were performed in a wide range of geographical settings leading to a diversity of racial groups. For the *Pst I/ Rsa I* polymorphisms, 9 studies examined individuals of Asians, 15 studies recruited Caucasians, one study in Brazilian [21], one study in Indian [16], and one study in Mexican [15]. For the *Dra I* polymorphism, all the studies were on Caucasians. In these studies, the genotype/allele distribution between ALD patients and the alcoholics without ALD, and/or between ALD patients and non-alcoholics, were compared. The detailed characteristics of the included studies were shown in the **Table 1** **and** **Table 2** **, respectively.** The detailed criteria for the selection of ALD patients, alcoholics without ALD, and non-alcoholics were shown in **Table S1**.

**Table 1:** Characteristics of individual studies for association between *CYP2E1 Pst I/Rsa I* polymorphisms and ALD risks.

Study	Country	Age^b^	Sex	Type of ALD	Cases			Alcoholics without ALD			Non-alcoholics			*P*_HWE_ d
					c1c1	c1c2	c2c2	c1c1	c1c2	c2c2	c1c1	c1c2	c2c2	
Liu,2012	China	51/na/na	Both	AC/AH/AFL/HCC	203	106	44	271	19	10	325	24	11	0.000
Garcia-Banuelos,2012	Mexico	44/42	Both	AC	25	16	0				73	15	2	0.266
Khan,2010	India	52/48/42	na	AC	161	14	0	137	3	0	250	5	0	0.874
Lorenzo,2006	Spain	55/54/48	Female	AC/AH/AFL	53	2	3	27	0	0	40	2	0	0.874
Cichoz-Lach,2006	Poland	50/44/51	Both	AC	53	4	0	43	0	0	54	0	0	na
Vidal,2004	Spain	53/57/46	Male	AC/AH/AFL	94	5	0	42	4	1	57	7	0	0.644
Kim,2004	Korea	49/49	Both	AC	17	4	0				51	34	15	0.029
Burim,2004	Brazil	18–76^c^	Both	AC	59	6	0	37	4	0	197	23	1	0.712
Kee,2003	Korea	na	na	AC	17	10	3	4	7	1	23	15	0	0.130
Frenzer,2002	Australia	58/42/50	Both	AC	56	1	0	54	3	0	188	12	0	0.662
Monzoni,2001	Italy	58/58	Both	AC/AH/AFL	66	14	1	85	7	0				na
Lee,2001	Korea	52/52/50	Male	AC	34	21	1	32	19	1	41	22	1	0.305
Zhang,2000	China	52.3^c^	Male	AC/AH/HCC	2	50	3				17	9	0	0.286
Wong,2000	UK	18–90^c^	Both	AH/AF/AC	59	2	0				350	25	0	0.504
Rodrigo,1999	Spain	55/60/45	Male	AC	112	8	0	28	2	0	183	17	0	0.530
Parsian,1998^a^	USA	na	Both	AC										na
Grove,1998	UK	na	Both	AC/AF/AH	226	14	0				117	4	0	0.853
Tanaka,1997	Japan	49/-	Male	AC/AF/AH	13	9	4	30	11	1				na
Chao,1997	China	51/50/22	Both	AC	42	29	4	12	5	2	56	38	6	0.894
Lucas,1996	France	55/44/(20–50)	na	AC	101	9	0	188	12	2	248	11	1	0.033
Carr,1996	China	55/40/20	na	AC	18	10	2	28	18	0	52	45	3	0.065
Agundez,1996	Spain	54/32	Both	AC	56	2	0				130	7	0	0.789
Yamauch,1995	Japan	(38–70)/-	Male	AC	34	12	0				40	18	2	0.989
Pirmohamed,1995	UK	na	Both	AC/AH	77	17	1	55	2	1	97	3	0	0.879
Carr,1995	USA	(33–72)/-/-	Male	AC/AH	49	3	1	35	4	0	31	1	0	0.929
Ball,1995	UK	na	na	AC	34	3	0				102	6	0	0.767
Ingelman-Sundberg,1993	Italy	na	na	AC	53	30	0				104	10	0	0.624

Abbreviations: ALD, alcoholic liver disease; AC, alcoholic cirrhosis; AH, alcoholic hepatisis; AFL, alcoholic fatty liver; AF, alcoholic fibrosis; HCC, hepatic carcinoma.

a the study by Parsian did not provide the number of each genotypes, instead they provided the number of alleles of case and controls;

b the mean age and/or the range of age of each groups;

c cases and controls combined;

d *p* for Hardy–Weinberg equilibrium test in controls (Healthy person);

“na“, means that the data were not available.

**Table 2:** Characteristics of individual studies for association between *CYP2E1 Dra I* polymorphisms and ALD risks.

Study	Country	Age^b^	Sex	Type ofALD	Cases			Alcoholics without ALD			Non-alcoholics			*P*_HWE_ d
					d2d2	d2d1	d1d1	d2d2	d2d1	d1d1	d2d2	d2d1	d1d1	
Khan,2009	India	52/49/42	na	AC	118	42	0	67	33	0	182	68	0	0.013
Lorenzo,2006	Spain	55/54/48	Female	AC/AH/AFL	36	22	0	18	9	0	34	8	0	0.495
Vidal,2004	Spain	53/57/46	Male	AC/AH/AFL	75	23	1	36	11	0	45	18	1	0.594
Frenzer,2002	Australia	58/42/50	Both	AC	48	9	0	46	10	1	170	28	2	0.489
Wong,2000	UK	18–90^c^	Both	AH/AF/AC	50	11	0				305	68	2	0.386
Parsian,1998^a^	USA	na	Both	AC										na
Savolainen,1997	Finland	35–69 c	Male	AH/AF/AC	156	48	3	30	6	0				na
Lucas,1996	France	55/44/(20–50)	na	AC	79	30	1	149	49	4	219	37	2	0.752
Ingelman-Sundberg,1993	Italy	na	na	AC	47	4	0				95	19	0	0.332

Abbreviations: ALD, alcoholic liver disease; AC, alcoholic cirrhosis; AH, alcoholic hepatisis; AFL, alcoholic fatty liver; AF, alcoholic fibrosis; HCC, hepatic carcinoma.

a the study by Parsian did not provide the number of each genotypes, instead they provided the number of alleles of case and controls;

b the mean age and/or the range of age of each groups;

c cases and controls combined;

d *p* for Hardy–Weinberg equilibrium test in controls (Healthy person);

“na“, means that the data were not available.

### Quantitative Data Synthesis

**Figure 2:**
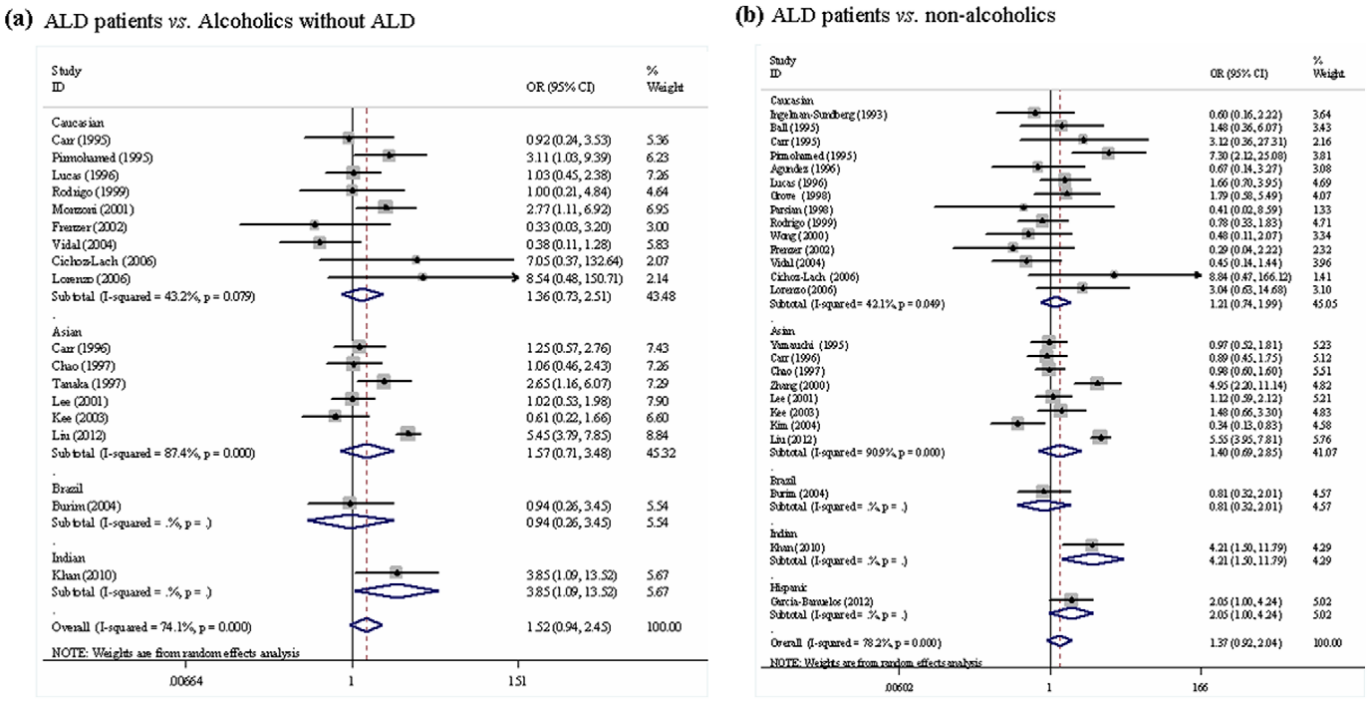
Meta-analysis for *CYP2E1 Pst I/Rsa I* polymorphism and the risk of ALD (allele c2 *vs.* c1). (a) ALD patients *vs.* alcoholics without ALD; (b) ALD patients *vs.* non-alcoholics. Each study was shown by a point estimate of the effect size (OR) (size inversely proportional to its variance) and its 95% confidence interval (95%CI) (horizontal lines). The white diamond denotes the pooled OR.

Results of pooled analysis on the associations between *CYP2E1 Pst I/Rsa I* polymorphism and the risk of ALD were shown in **Table S1**. Overall, no significant association between c2 allele and the risks of ALD was observed (ALD patients *vs.* alcoholics without ALD: OR = 1.52, 95%CI 0.94–2.46; ALD patients *vs.* non-alcoholics: OR = 1.37, 95%CI 0.92–2.04) (**Figure 2**). However, a significant association was observed in the homozygous genotype comparison (c2c2 *vs.* c1c1: OR = 3.12, 95%CI 1.91–5.11) when ALD patients were compared with alcoholics without ALD, which was not observed in other genotypes contrasts. In regard with the genotypes contrast between ALD patients and non-alcoholics, no significant association was detected in any genetic model (c2c2 *vs.* c1c1: OR = 1.83, 95%CI 0.80–4.21; c2c2/c2c1 *vs.* c1c1: OR = 1.48, 95%CI 0.93−2.34).

**Figure 3:**
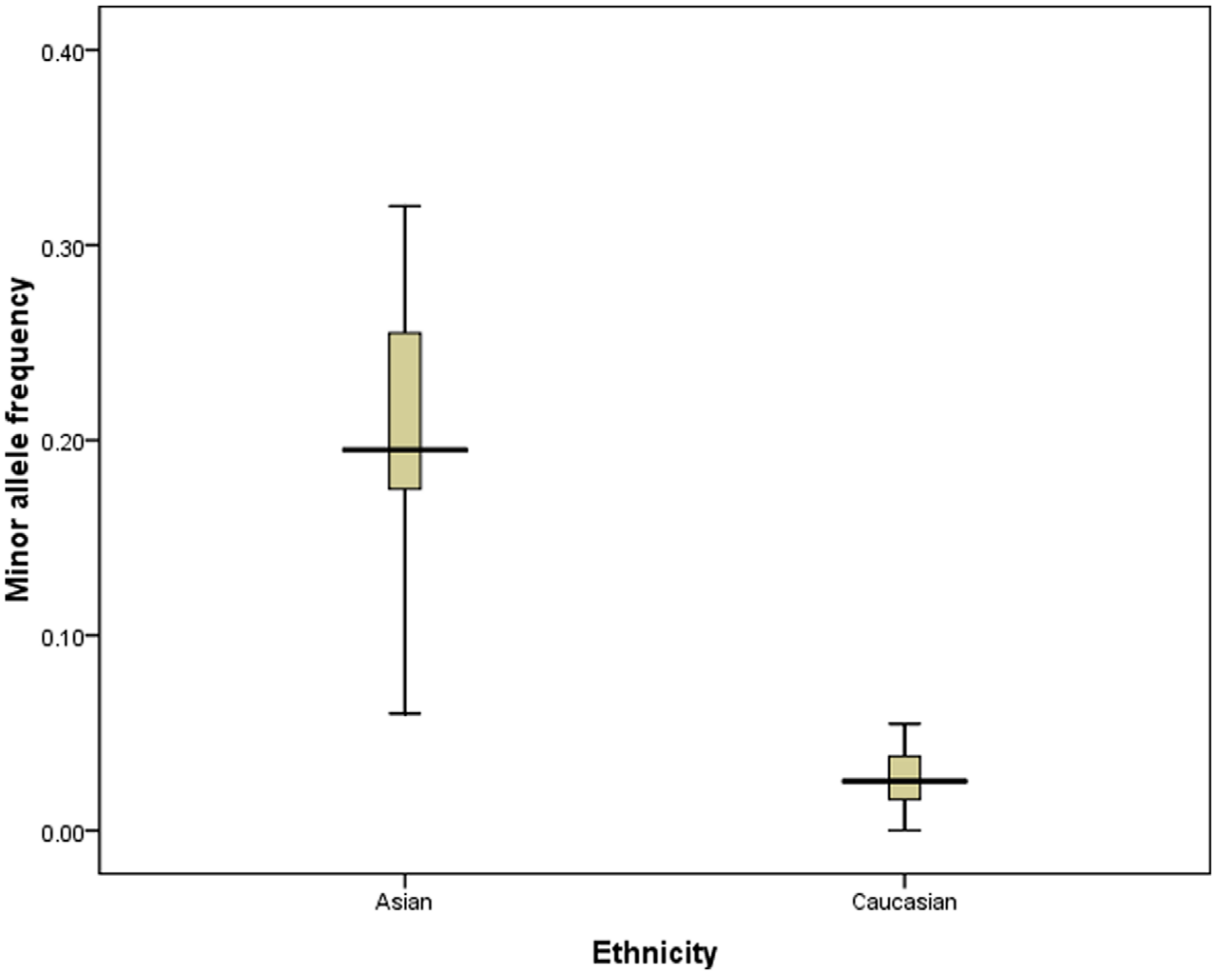
Frequencies of the minor allele (c2 allele) of the *CYP2E1 Pst I/Rsa I* polymorphism among control subjects stratified by ethnicity.

Due to the ethnicity-related distinct discrepancy of allele distribution in non-alcoholics (20.4% and 2.7% in Asians and in Caucasians, respectively) (**Figure 3**), we then made subgroup analysis based on ethnicity. The results revealed that c2c2 genotype was also significantly associated with increased risk of ALD in Asians (c2c2 *vs.* c1c1: OR = 4.11, 95%CI 2.32–7.29), while significant associations were also observed in Caucasians (c1c2 *vs.* c1c1: OR = 1.63, 95%CI 1.05–2.53; c2c2/c1c2 *vs.* c1c1: OR = 1.58, 95%CI 1.04–2.42) when ALD patient were compared with alcoholics without ALD. However, no significant association was also observed in Asians or Caucasians when ALD patients were compared with no-alcoholics (**Table S2**).

As ALD contains many types of histological forms, including steatosis, hepatisis, fibrosis, and cirrhosis, we then investigated the associations between *CYP2E1 Pst I/Rsa I* polymorphism and the risks of clearly defined alcoholic liver cirrhosis (ALC). As shown in **Table S3**, no significant association was observed between *CYP2E1 Pst I/Rsa I* polymorphism and ALC risks in Asians (c2 *vs.* c1, OR = 1.00, 95%CI 0.67–1.49; c2c2/c2c1 *vs.* c1c1, OR = 0.97, 95%CI 0.60–1.57) as well as in Caucasians (c2 *vs.* c1, OR = 1.06, 95%CI 0.63–1.79; c2c2/c2c1 *vs.* c1c1, OR = 1.19, 95%CI 0.69–2.06) when ALC patients were compared with alcoholics without ALD. However, the polled results of the studies, in which cases were composed by several types of ALD patients including steatosis, hepatisis, fibrosis, and cirrhosis, showed significant associations in Asians (c2 *vs.* c1, OR = 4.95, 95%CI 3.55–6.89; c2c2/c2c1 *vs.* c1c1, OR = 4.63, 95%CI 1.75–12.26) and in Caucasians (c2 *vs.* c1, OR = 2.58, 95%CI 1.42–4.67; c2c2/c2c1 *vs.* c1c1, OR = 2.58, 95%CI 1.37–4.87) (**Table S3**).

To investigate whether there was difference in the association between *CYP2E1 Pst I/Rsa I* polymorphisms and ALD risks in men and women, we then combined the results of studies in which only male subjects were enrolled. Meta-analysis for the association between CYP2E1 *Pst I/Rsa I* polymorphism and the risk of ALD in male subjects were shown in **Table S4**. As shown in **Table S4**, no significant association was detected in any comparisons.

**Figure 4:**
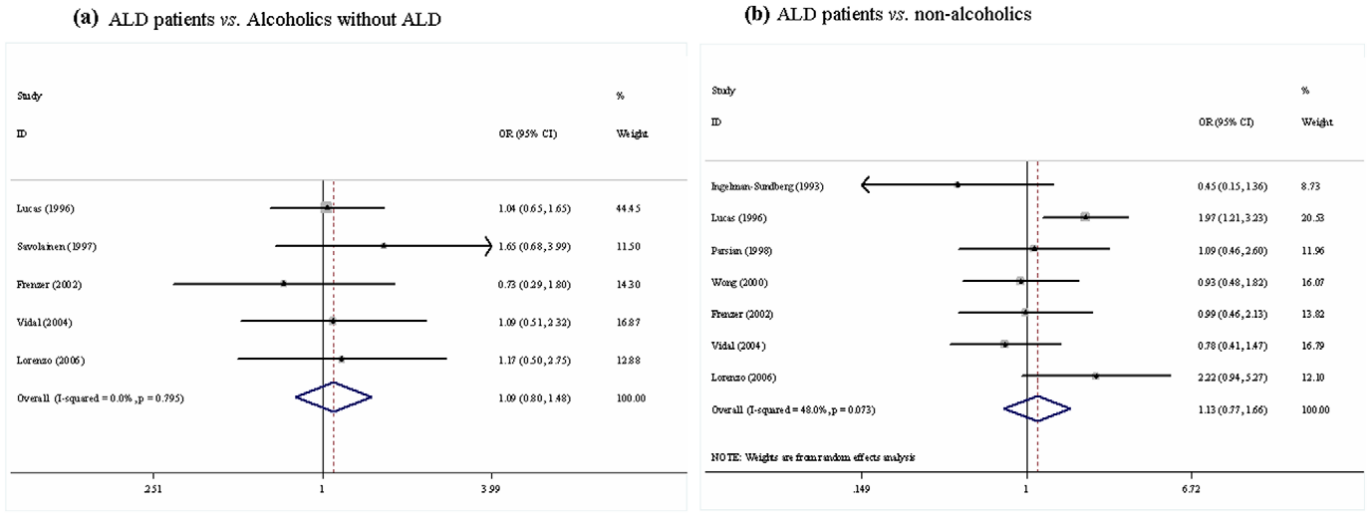
Meta-analysis for *CYP2E1 Dra I* polymorphism and the risk of ALD in Caucasians (d1 *vs.* d2). (a) ALD patients *vs.* alcoholics without ALD; (b) ALD patients *vs.* non-alcoholics. Each study was shown by a point estimate of the effect size (OR) (size inversely proportional to its variance) and its 95% confidence interval (95%CI) (horizontal lines). The white diamond denotes the pooled OR.

Results of pooled analysis on the associations between *CYP2E1 Dra I* polymorphism and the risk of ALD were shown in **Table S5 and** **Figure 4**. The combined results showed no significant association between *CYP2E1 Dra I* polymorphism and the risk of ALD (ALD patients *vs.* alcoholics without ALD: d1 *vs.* d2, OR = 1.09, 95%CI 0.80–1.48; d1d1/d1d2 *vs*. d2d2, OR = 1.00, 95% CI 0.75–1.33; ALD patients *vs.* non-alcoholics: d1 *vs.* d2, OR = 1.13, 95%CI 0.77–1.66; d1d1/d1d2 *vs.* d2d2: OR = 1.13, 95% CI 0.76–1.70). We further analyzed the relationship between *CYP2E1 Dra I* polymorphism and the risks of ALC. Again, the pooled results revealed no significant association (**Table S5**).

### Heterogeneity Source and Sensitivity Analysis

The between-study heterogeneity was significant in the analyses of association between *CYP2E1 Pst I/Rsa I* polymorphism and the risk of ALD in worldwide population (*I^2^>50% in most comparisons*). Subgroup meta-analysis has been used as a common method for exploring the heterogeneity [41]. The heterogeneity test of the subgroup of Asians and Caucasians showed much lower heterogeneity existed in the analyses of association between *CYP2E1 Pst I/Rsa I or Dra I* polymorphisms and the risk of ALD in Caucasians, which suggested that ethnicity might be an important contributor to heterogeneity. The heterogeneity test in the subgroup analyses based on the types of ALD showed that lower heterogeneity in the analysis of the association between *CYP2E1 Pst I/Rsa I* polymorphism and ALC risks, indicating that types of ALD might be another source of the between study heterogeneity. Sensitivity analysis was performed by sequential omission of individual studies in every comparison, and the data showed that no study significantly influenced the pooled effects by omitting any single study. For the comparisons between ALD patients and non-alcoholics, the exclusion of the studies deviated form HWE did not change the results significantly.

### Publication Bias

Begg’s funnel plots were generated to assess publication bias. The Egger’s test was performed to statistically evaluate funnel plot symmetry. Potential publication bias was detected by Egger’s test for the comparisons between c2 *vs.* c1, c1c2 *vs.* c1c1, and c2c2/c2c1 *vs.* c1c1 (*P* = 0.025, 0.036, and 0.020, respectively) when ALD patients were compared with alcoholics without ALD in Asians. No significant publication bias was detected in any other comparisons (P_value_ *>* 0.05 in both Egger’s regression and Begg’s rank correlation tests).

## Discussion

ALD is a multifactorial process involving several mechanisms, in which oxidative stress may play an important role [9]. Many pathways have been suggested to be associated with ethanol-induced oxidative stress. Among them, the activation of CYP2E1 appears to be a major contributor. It has been found that ethanol -induced liver injury and lipid peroxidation was correlated well with the CYP2E1 levels [42]–[44]. Furthermore, CYP2E1 inhibitors significantly blocked the lipid peroxidation and ameliorated the pathologic changes in ethanol-treated animals [1], [8], [45], [46], while CYP2E1 over-expressing mice displayed higher transaminase activities and histological features of liver injury when compared with the control mice [47]. All these studies support the critical roles of CYP2E1 in the etiology of ALD. Thus, the *CYP2E1* functional polymorphisms including *Pst I/Rsa I or Dra I* polymorphisms might be associated with the ALD risks of individuals. Unfortunately, previous epidemiological studies conducted in the past 20 decades have yielded conflicting results, ranging from strong association to no links. Because of the above-mentioned conflicting results from relatively small sample size which might be underpowered to detect the potential effects, a meta-analysis might be an appropriate approach to obtain a more definitive conclusion.

In the current study, we made a comprehensive literature search and a total of 27 and 9 studies were finally included for the analysis of the associations between the *CYP2E1 Pst I/Rsa I* polymorphisms and the risk of ALD, and between *CYP2E1 Dra I* polymorphism and the risk of ALD, respectively. The pooled results showed that c2c2 genotype was significantly associated with increased risk of ALD in Asians (**Table S2**). These data were well consistent with many previous studies [14], [29], [48]. Another important finding was that a significant association between the *CYP2E1 Pst I/Rsa I* polymorphism and the ALD risk was also found in Caucasians (c1c2 *vs.* c1c1: OR = 1.63, 95%CI 1.05–2.53; c2c2/c1c2 *vs.* c1c1: OR = 1.58, 95%CI 1.04–2.42), although many pervious studies reported no association [11], [17], [19], [21], [23], [26], [27], [33], [36], [38]. The relatively small sample size in individual studies might cover the potential association, as the frequency of c2 allele in Caucasians was much lower than that in Asians (20.4% *vs.* 2.7% in Asians and Caucasians, respectively) (**Figure 3**). In contrast to the significant association between *CYP2E1 Pst I/Rsa I* polymorphism and the risk of ALD, no significant association was observed between *CYP2E1 Dra I* polymorphism and the risk of ALD, although the frequency of the *Dra I* polymorphism was about 3-fold of that of the *Pst I/Rsa I* polymorphism in Caucasians (2.7% *vs.* 9.8% in Caucasians in this study) [49].

Zintzaras *et al.* performed a meta-analysis to evaluate the association between the polymorphisms of ethanol-metabolizing enzymes including *CYP2E1 Pst I/Rsa I* polymorphism and the risk of ALD [50]. In that study, 8 studies were included for the association assay. The authors evaluated the association of the mutant allele with ALC risks, and the results showed that c2 allele was not significantly associated with the ALC risks (worldwide population: OR = 1.13, 95%CI 0.76–1.68; Caucasians: OR = 1.58, 95%CI 0.76–3.28; Asians: OR = 0.97, 95%CI 0.58–1.62). In the current study, a total of 27 studies were included in the analysis, and the combined results showed a significant association in the genotype contrasts in Asians (c2c2 *vs.* c1c1, OR = 4.11, 95%CI 2.32–7.29) as well as in Caucasians (c2c2/c1c2 *vs.* c1c1, OR = 1.58, 95%CI 1.04–2.42) (**Table S2**). However, the subgroup analysis on the association between *CYP2E1 Pst I/Rsa I* polymorphism and ALC risks in the current meta-analysis showed similar results to those obtained in the study by Zintzaras *et al.* (Caucasians: OR = 1.26, 95%CI 0.56–2.85; Asians: OR = 1.00, 95%CI 0.67–1.49) (**Table S3**). These results suggested that *CYP2E1 Pst I/Rsa I* polymorphism might be not significantly associated with the ALC risks, but might be significantly associated with other types of ALD. In fact, ALD represents a spectrum of clinical illness and morphological changes that range from alcoholic fatty liver (AFL) to hepatic inflammation and necrosis (alcoholic hepatitis, AH) to progressive fibrosis (AF) and cirrhosis [51]. It may be speculated that the *CYP2E1 Pst I/Rsa I* polymorphism might be significantly associated with the earlier liver damages such as AFL and AH, but not with the advanced ALD (ALC), which need to be evaluated in the future studies. This meta-analysis firstly addressed the association between *CYP2E1 Dra I* polymorphism and the risk of ALD, but no significant association was found based on 9 studies performed in Caucasians.

One of the most characteristic features that distinguish CYP2E1 from other ethanol metabolizing enzymes is its inducibility. Some studies demonstrated that ethanol could prevent the decomposition of CYP2E1 protein without influencing the mRNA levels [52], [53]; while other studies proposed that ethanol could activate CYP2E1 synthesis [54]. It is possible that transcriptional, posttranscriptional and posttranslational events may all take effects in ethanol-induced activation of CYP2E1. As ethanol could activate CYP2E1 by stabilization or increasing synthesis, the mRNA levels are not systematically enhanced. Therefore, detection of CYP2E1 activity *in vivo* would be a more reliable method for the evaluation of CYP2E1 status, and may be helpful to evaluate the association of *CYP2E1* polymorphisms and the risk of ALD. If subjects with c2c2/c1c2 genotypes have higher CYP2E1 activity, then a solid conclusion about the association between *CYP2E1 Pst I/Rsa I* polymorphism and ALD risk, would be more rational and reliable, as these persons would be more susceptive to ALD. However, this information is limited in the literature. A preliminary study on the elimination of acetaminophen, which was metabolized mainly by CYP2E1, suggested that individuals with homozygous c2c2 genotypes exhibited higher CYP2E1 activity than individuals with c1c1 or c1c2 genotypes [55]. However, several other studies in which the activity of CYP2E1 was detected using chlorzoxazone metabolism have revealed that subjects with c2 alleles did not have enhanced CYP2E1 activity [56], [57]. Anyway, much more studies were still needed to clarify the relationship between the *CYP2E1 Pst I/Rsa I* polymorphisms and the activity of CYP2E1 *in vivo*.

Despite clear strengths of our study including the larger sample size and comprehensive literature search, it dose have some limitations. Firstly, the present meta-analysis was based on unadjusted effect estimates and CIs, since most studies did not provide the adjusted OR and 95%CI controlling for potential confounding factors. Secondly, moderate to higher heterogeneity existed for the analyses especially for the subgroup of Asians. Thirdly, it has been well known that ALD is a multifactor diseases, however, the effects of gene-gene and gene-environment interactions were not addressed in this meta-analysis, and thus the potential roles of the above gene polymorphism may be masked or magnified by other gene-gene/gene-environment interactions.

In summary, this meta-analysis systematically analyzed the association between the *CYP2E1 Pst I/Rsa I* and *Dra I* polymorphisms and the risk of ALD. The combined results showed that *CYP2E1 Pst I/Rsa I* polymorphism might be not significantly associated with advanced form of ALD (ALC), but might be significantly associated with other form of ALD such as steatosis, hepatisis, fibrosis. Furthermore, *CYP2E1 Dra I* polymorphism might be not significantly associated with ALD risks. Since potential confounders could not be ruled out completely, further studies are needed to confirm these results.

## Supporting Information

Table S1 The criteria for selection of cases and controls of included studies.
(DOC)

Table S2 Meta-analysis for the association between *CYP2E1 Pst I/Rsa I* polymorphism and the risk of ALD.
(DOC)

Table S3 Subgroup analysis on the association between *CYP2E1 Pst I/Rsa I* polymorphism and the risk of ALD
(DOC)

Table S4 Meta-analysis for the association between *CYP2E1 Pst I/Rsa I* polymorphism and the risk of ALD in male subjects
(DOC)

Table S5 Meta-analysis for the association between *CYP2E1 Dra I* polymorphism and the risk of ALD
(DOC)

PRISMA Checklist S1 PRISMA Checklist.
(DOC)
