# Supplementary material for: Roles of Cytochrome P4502E1 Gene Polymorphisms and the Risks of Alcoholic Liver Disease: A Meta-Analysis
Source: PLoS One. 2013 Jan 15;8(1):e54188. doi: 10.1371/journal.pone.0054188 (PMC3545986; doi:10.1371/journal.pone.0054188)
Supplement: Table S1 — The criteria for selection of cases and controls of included studies. (DOC) [file pone.0054188.s001.doc]

**Table S1 the criteria for selection of cases and controls of included studies**

| **Study ID** | **The criteria for selection of cases and controls** |
| --- | --- |
| Liu,2012 | **Cases**: including AC, AH, and AFL; alcohol intake *>* 40 g/d for women and 80 g/d for men for at least 5 years;  **Alcoholics without ALD**: alcohol dependent without ALD;  **Non-alcoholics**: healthy people. |
| Garcia-Banuelos,2012 | **Cases**: alcohol intake *>* 40 g/d for more than 4 years; liver cirrhosis was based on clinical, biochemical, and histological criteria;  **Non-alcoholics**: clinically healthy control individuals whose alcohol intake was null or sporadic. |
| Khan,2010 | **Cases**: AC was diagnosed on the basis of patient’s history of alcohol intake provided by relevant family members.  **Alcoholics without ALD**: alcohol intake *>* 80 g/d for more than 10 years; without liver disease diagnosed by physical examination and liver function test;  **Non-alcoholics**: alcohol intake *<* 10 g/d; without liver disease diagnosed by physical examination and liver function test. |
| Khan, 2009 | **Cases**: alcohol intake *>* 80 g/d for more than 10 years; AC was diagnosed on the basis of their liver biopsy;  **Alcoholics without ALD**: alcohol intake *>* 80 g/d for more than 10 years; without liver disease diagnosed by physical examination and liver function test;  **Non-alcoholics**: alcohol intake *<* 10 g/d; without liver disease diagnosed by physical examination and liver function test. |
| Lorenzo,2006 | **Cases**: alcoholics was defined according to the CAGE (acronym of Cut, Annoyed, Guilty, Eye opener) questionnaire; the different types of ALD were diagnosed by liver biopsy;  **Alcoholics without ALD**: histological examination indicated that the liver was normal;  **Non-alcoholics**: healthy controls with no history of alcoholism, no evidence of liver disease. |
| Cichoz-Lach,2006 | **Cases**: alcohol intake *>* 80 g/d for at least 2 years; the diagnosis of AC was based on the medical history, physical examination, liver function test, imaging examinations-ultrasound of the abdominal cavity, computed tomography of the abdominal cavity, doppler ultrasound, and liver biopsy;  **Alcoholics without ALD**: alcohol intake *>* 80 g/d for at least 2 years; liver function tests were normal;  **Non-alcoholics**: healthy complete nondrinkers. |
| Vidal,2004 | **Cases**: alcohol intake *>* 100 g/d for more than 10 years; the different types of ALD were diagnosed by liver biopsy;  **Alcoholics without ALD**: alcohol intake *>* 100 g/d for more than 10 years; histological examination indicated a normal liver;  **Non-alcoholics**: alcohol intake *<* 10 g/d; with no history of alcoholism, no evidence of liver disease at physical examination and liver function tests. |
| Kim,2004 | **Cases**: the definition of AC could not be obtained as this study was published in Korean;  **Non-alcoholics**: Healthy blood donor. |
| Burim,2004 | **Cases**: alcohol intake *>* 40 g/d; the diagnoses of cirrhosis were based on clinical, biochemical and echographic examination;  **Alcoholics without ALD**: alcohol intake *>* 40 g/d; exclusion of ALD based on clinical, biochemical and echographic examination;  **Non-alcoholics**: general population. |
| Kee,2003 | **Cases**: the definition of AC could not be obtained as this study was published in Korean;  **Alcoholics without ALD**: the definition of Alcoholics without ALD could not be obtained as this study was published in Korean;  **Non-alcoholics**: healthy control. |
| Frenzer,2002 | **Cases**: AC was diagnosed by liver biopsy or had unequivocal clinical, radiological and endoscopic features of cirrhosis;  **Alcoholics without ALD**: recruited from a detoxification center for alcoholics without clinical features of chronic liver disease;  **Non-alcoholics**: healthy blood donor recruited from a blood bank. |
| Monzoni,2001 | **Cases**: heavy drinker with cirrhosis or with persistent levels of either ALT or AST or GGT 1.5-fold as the upper normal range in at least six out of the eight 6-month interval checks and who showed signs of steatosis at ultrasonography;  **Alcoholics without ALD**: heavy drinkers without sings of ALD. |
| Lee,2001 | **Cases**: alcohol intake *>* 80 g/d for more than 10 years; have clinical evidences of portal hypertension such as esophageal varices, ascites, or hepatic encephalopathy.  **Alcoholics without ALD**: alcohol intake *>* 80 g/d for more than 10 years; had normal liver function test values without clinical or radiological (ultrasonography or CT) evidence of liver disease;  **Non-alcoholics**: age-matched healthy males who drank less than 80 g/week. |
| Zhang,2000 | **Cases**: alcohol intake *>* 80 g/d for more than 10 years; the diagnoses of ALD were based on clinical, biochemical and echographic and histological examination;  **Non-alcoholics**: general population. |
| Wong,2000 | **Cases**: alcohol intake *>* 60 g/d for more than 10 years; Diagnoses of ALD were based on liver biopsies;  **Non-alcoholics**: healthy person. |
| Rodrigo,1999 | **Cases**: alcohol intake *>* 200 g/d for at least 20 years; the diagnoses of cirrhosis were based on clinical and histological test;  **Alcoholics without ALD**: healthy heavy drinkers with alcohol intake *>* 200 g/d for more than 30 years;  **Non-alcoholics**: healthy people. |
| Parsian,1998 | **Cases**: AC was diagnosed by histological examination;  **Non-alcoholics**: person did met DSM-III-R criteria for affective disorders, alcoholism, schizophrenia, psychotic/ drug use disorders. |
| Grove,1998 | **Cases**: alcohol intake *>* 80 g/d for more than 10 years; diagnoses of ALD were based on clinical, biochemical and liver biopsy;  **Non-alcoholics**: hospital and university staff with alcohol intake *<* 210 g/week for men or 140 g/week for women, and had normal liver blood tests (ALT, ALP, bilirubin). |
| Tanaka,1997 | **Cases**: alcohol intake *>* 80 g/d for more than 10 years; diagnoses of ALD were based on liver function tests and liver biopsy;  **Alcoholics without ALD**: general population with alcohol intake *>* 80 g/d. |
| Savolainen,1997 | **Cases**: alcohol intake *>* 80 g/d; diagnoses of ALD were based on autopsy;  **Alcoholics without ALD**: alcohol intake *>* 80 g/d, exclusion of ALD was based on autopsy. |
| Chao,1997 | **Cases:** alcohol intake *>* 60 g/d for at least 7 years; the diagnoses of cirrhosis were based on typical sonographic signs;  **Alcoholics without ALD**: alcohol intake *>* 60 g/d for at least 7 years; without liver diseases according to sonographic, endoscopical, and biochemical studies;  **Non-alcoholics**: male medical students who did not have history of alcoholism and did not usually drink alcoholic beverages. |
| Lucas,1996 | **Cases**: mean alcohol intake was 148 g/d for 28 years; the diagnoses of cirrhosis were based on histological examination;  **Alcoholics without ALD**: mean alcohol intake was 193 g/d for17 years; without clinical symptoms of liver diseases;  **Non-alcoholics**: research staff and medical students. |
| Carr,1996 | **Cases**: male AC patients with alcohol intake *>* 80 g/d for more than 10 years;  **Alcoholics without ALD**: male alcohol-dependent patients without liver complication;  **Non-alcoholics:** students from the National Defense Medical Center in Taipei. |
| Agundez,1996 | **Cases**: alcohol intake *>* 100g/d for more than 10 years; AC was diagnosed by clinical, ultrasonographic and histological examination;  **Non-alcoholics**: healthy volunteers. |
| Yamauch,1995 | **Cases**: alcohol intake *>* 120 g/d for more than 10 years; cirrhosis was diagnosed based on liver biopsy;  **Non-alcoholics**: unrelated, age-matched, male healthy subjects. |
| Pirmohamed,  1995 | **Cases**: excessive alcohol intake (median 190 g/d) for a prolonged period (median 10 years); the diagnoses of cirrhosis were based on clinical signs and symptoms, and deranged biochemical liver function tests, and liver biopsy;  **Alcoholics without ALD**: excess alcohol intake (median 216 g/d) for prolonged periods (median 12 years); without any clinical or biochemical evidence of liver disease;  **Non-alcoholics**: healthy volunteers. |
| Carr,1995 | **Cases**: excessive alcohol intake (median 257 g/d) for a prolonged period (median 27 years); the diagnoses of AH and AC were based on histological examination;  **Alcoholics without ALD**: Control subjects included 21 alcoholics without liver disease (liver function test in normal level);  **Non-alcoholics**: the staff and employees of the Indiana University School of Medicine. |
| Ball,1995 | **Cases**: alcohol intake *>* 80 g/d for more than 3 years; AC was diagnosed based on liver biopsy;  **Non-alcoholics**: Ethnically-matched population controls. |
| Ingelman-Sundberg,1993 | **Cases**: alcoholics with clinical signs of cirrhosis;  **Non-alcoholics**: Italy controls. |

Abbreviations: ALD, alcoholic liver disease; AC, alcoholic cirrohiss; AH, alcoholic hepatisis; AFL, alcoholic fatty liver; AF, alcoholic fibrosis.
