# Supplementary material for: Roles of Cytochrome P4502E1 Gene Polymorphisms and the Risks of Alcoholic Liver Disease: A Meta-Analysis
Source: PLoS One. 2013 Jan 15;8(1):e54188. doi: 10.1371/journal.pone.0054188 (PMC3545986; doi:10.1371/journal.pone.0054188)
Supplement: Table S2 — Meta-analysis for the association between CYP2E1 Pst I/Rsa I polymorphism and the risk of ALD. (DOC) [file pone.0054188.s002.doc]

**Table S2 Meta-analysis for the association between** ***CYP2E1 Pst I/Rsa I* polymorphism and the risk of ALD**

|  |  | Contrasts | No. of studies | Test of association | | | |  | Test of heterogeneity | |  | *P*_Egger’s test_ ^c^ |
| --- | --- | --- | --- | --- | --- | --- | --- | --- | --- | --- | --- | --- |
|  |  |  |  | OR | 95%CI | M ^a^ | *P*_OR_ |  | *I*^2^ (%) | *P*_value_^b^ |  |  |
| All | ALD patients  *vs.*  Alcoholics without ALD | c2 vs. c1 | 17 | 1.52 | 0.94-2.46 | R | 0.086 |  | 74.1 | 0.000 |  | 0.325 |
|  |  | c2c2 vs. c1c1 | 12 | 3.12 | 1.91-5.11 | F | 0.000 |  | 31.6 | 0.138 |  | 0.127 |
|  |  | c1c2 vs. c1c1 | 17 | 1.53 | 0.90-2.59 | R | 0.114 |  | 69.0 | 0.000 |  | 0.199 |
|  |  | c2c2+c1c2 vs. c1c1 | 17 | 1.56 | 0.92-2.63 | R | 0.099 |  | 72.4 | 0.000 |  | 0.184 |
|  | ALD patients  *vs.* Non-alcoholics | c2 vs. c1 | 25 | 1.37 | 0.92-2.04 | R | 0.120 |  | 78.2 | 0.000 |  | 0.272 |
|  |  | c2c2 vs. c1c1 | 14 | 1.83 | 0.80-4.21 | R | 0.153 |  | 46.1 | 0.030 |  | 0.429 |
|  |  | c1c2 vs. c1c1 | 24 | 1.44 | 0.91-2.26 | R | 0.116 |  | 77.1 | 0.000 |  | 0.587 |
|  |  | c2c2+c1c2 vs. c1c1 | 24 | 1.48 | 0.93-2.34 | R | 0.097 |  | 79.6 | 0.000 |  | 0.647 |
| Asian | ALD patients  *vs.*  Alcoholics without ALD | c2 vs. c1 | 6 | 1.57 | 0.71-3.49 | R | 0.265 |  | 87.4 | 0.000 |  | 0.025 |
|  |  | c2c2 vs. c1c1 | 6 | 4.11 | 2.32-7.29 | F | 0.000 |  | 45.0 | 0.105 |  | 0.437 |
|  |  | c1c2 vs. c1c1 | 6 | 1.49 | 0.56-3.97 | R | 0.428 |  | 85.5 | 0.000 |  | 0.036 |
|  |  | c2c2+c1c2 vs. c1c1 | 6 | 1.57 | 0.61-4.01 | R | 0.351 |  | 86.4 | 0.000 |  | 0.020 |
|  | ALD patients  *vs.* Non-alcoholics | c2 vs. c1 | 8 | 1.40 | 0.69-2.86 | R | 0.349 |  | 90.9 | 0.000 |  | 0.127 |
|  |  | c2c2 vs. c1c1 | 8 | 1.87 | 0.57-6.12 | R | 0.304 |  | 68.0 | 0.003 |  | 0.596 |
|  |  | c1c2 vs. c1c1 | 8 | 1.66 | 0.69-3.99 | R | 0.259 |  | 89.6 | 0.000 |  | 0.921 |
|  |  | c2c2+c1c2 vs. c1c1 | 8 | 1.68 | 0.69-4.09 | R | 0.257 |  | 91.1 | 0.000 |  | 0.836 |
| Caucasians | ALD patients  *vs.*  Alcoholics without ALD | c2 vs. c1 | 9 | 1.36 | 0.73-2.51 | R | 0.330 |  | 43.2 | 0.079 |  | 0.853 |
|  |  | c2c2 vs. c1c1 | 6 | 1.06 | 0.36-3.08 | F | 0.919 |  | 0.0 | 0.640 |  | 0.879 |
|  |  | c1c2 vs. c1c1 | 9 | 1.63 | 1.05-2.53 | F | 0.031 |  | 31.0 | 0.170 |  | 0.879 |
|  |  | c2c2+c1c2 vs. c1c1 | 9 | 1.58 | 1.04-2.42 | F | 0.033 |  | 37.6 | 0.118 |  | 0.966 |
|  | ALD patients  *vs.* Non-alcoholics | c2 vs. c1 | 13 | 1.21 | 0.74-1.99 | R | 0.455 |  | 42.1 | 0.049 |  | 0.902 |
|  |  | c2c2 vs. c1c1 | 4 | 2.61 | 0.61-11.24 | F | 0.197 |  | 0.0 | 0.848 |  | 0.348 |
|  |  | c1c2 vs. c1c1 | 13 | 1.13 | 0.68-1.88 | R | 0.643 |  | 41.6 | 0.058 |  | 0.969 |
|  |  | c2c2+c1c2 vs. c1c1 | 13 | 1.20 | 0.72-2.00 | R | 0.489 |  | 43.3 | 0.048 |  | 0.808 |
| Brazil | ALD patients  *vs.*  Alcoholics without ALD | c2 vs. c1 | 1 | 0.94 | 0.26-3.45 | F | 0.930 |  | — | — |  | — |
|  |  | c2c2+c1c2 vs. c1c1 | 1 | 0.94 | 0.25-3.56 | F | 0.928 |  | — | — |  | — |
| Mexican | ALD patients  *vs.* Non-alcoholics | c2 vs. c1 | 1 | 2.05 | 1.00-4.24 | F | 0.051 |  | — | — |  | — |
|  |  | c2c2+c1c2 vs. c1c1 | 1 | 2.75 | 1.21-6.24 | F | 0.016 |  | — | — |  | — |
| Indian | ALD patients  *vs.*  Alcoholics without ALD | c2 vs. c1 | 1 | 3.85 | 1.10-13.52 | F | 0.036 |  | — | — |  | — |
|  |  | c2c2+c1c2 vs. c1c1 | 1 | 3.97 | 1.12-14.11 | F | 0.033 |  | — | — |  | — |

^a^ Model, model of meta-analysis; F, fixed effect model; R, random effect model.

^b^ *P*_value_*,*, *P* value for heterogeneity based on Q test.

^c^ *P*_Egger’s_ _test_*, P* value for Egger’s test.

“—” Values could not be calculated out.
