# Supplementary material for: Roles of Cytochrome P4502E1 Gene Polymorphisms and the Risks of Alcoholic Liver Disease: A Meta-Analysis
Source: PLoS One. 2013 Jan 15;8(1):e54188. doi: 10.1371/journal.pone.0054188 (PMC3545986; doi:10.1371/journal.pone.0054188)
Supplement: Table S3 — Subgroup analysis on the association between CYP2E1 Pst I/Rsa I polymorphism and the risk of ALD (DOC) [file pone.0054188.s003.doc]

**Table S3 Subgroup analysis on the association between *CYP2E1 Pst I/Rsa I polymor*phism and the risk of ALD**

|  |  | Contrasts | No. of studies | Test of association | | | |  | Test of heterogeneity | |  | *P*_Egger’s test_ ^c^ |
| --- | --- | --- | --- | --- | --- | --- | --- | --- | --- | --- | --- | --- |
|  |  |  |  | OR | 95%CI | M ^a^ | *P*_OR_ |  | *I*^2^ (%) | *P*_value_^b^ |  |  |
| Asians | ALC patients  *vs.*  Alcoholics without ALD | c2 vs. c1 | 4 | 1.00 | 0.67-1.49 | F | 0.999 |  | 0.0 | 0.733 |  | 0.352 |
|  |  | c2c2 vs. c1c1 | 4 | 1.15 | 0.36-3.62 | F | 0.813 |  | 0.0 | 0.534 |  | 0.238 |
|  |  | c1c2 vs. c1c1 | 4 | 0.95 | 0.58-1.56 | F | 0.827 |  | 0.0 | 0.399 |  | 0.498 |
|  |  | c2c2+c1c2 vs. c1c1 | 4 | 0.97 | 0.60-1.57 | F | 0.896 |  | 0.0 | 0.546 |  | 0.309 |
|  | Other cases  *vs.*  Alcoholics without ALD | c2 vs. c1 | 2 | 4.95 | 3.55-6.89 | F | 0.000 |  | 59.2 | 0.118 |  | — |
|  |  | c2c2 vs. c1c1 | 2 | 6.10 | 3.09-12.02 | F | 0.000 |  | 0.0 | 0.711 |  | — |
|  |  | c1c2 vs. c1c1 | 2 | 4.09 | 1.08-15.59 | R | 0.039 |  | 79.8 | 0.026 |  | — |
|  |  | c2c2+c1c2 vs. c1c1 | 2 | 4.63 | 1.75-12.26 | R | 0.002 |  | 69.1 | 0.072 |  | — |
|  | ALC patients  *vs.*  Non-alcoholics | c2 vs. c1 | 6 | 0.87 | 0.67-1.14 | F | 0.322 |  | 30.2 | 0.208 |  | 0.343 |
|  |  | c2c2 vs. c1c1 | 6 | 0.85 | 0.41-1.78 | F | 0.674 |  | 12.6 | 0.334 |  | 0.829 |
|  |  | c1c2 vs. c1c1 | 6 | 0.83 | 0.59-1.16 | F | 0.279 |  | 0.0 | 0.615 |  | 0.070 |
|  |  | c2c2+c1c2 vs. c1c1 | 6 | 0.83 | 0.60-1.15 | F | 0.265 |  | 3.6 | 0.394 |  | 0.209 |
|  | Other cases  *vs.*  Non-alcoholics | c2 vs. c1 | 3 | 3.00 | 0.96-9.36 | R | 0.059 |  | 91.5 | 0.000 |  | 0.600 |
|  |  | c2c2 vs. c1c1 | 3 | 4.01 | 0.33-48.73 | R | 0.276 |  | 71.2 | 0.031 |  | 0.605 |
|  |  | c1c2 vs. c1c1 | 3 | 6.41 | 1.29-31.95 | R | 0.023 |  | 91.4 | 0.000 |  | 0.440 |
|  |  | c2c2+c1c2 vs. c1c1 | 3 | 6.23 | 1.21-32.13 | R | 0.029 |  | 92.6 | 0.000 |  | 0.482 |
| Caucasians | ALC patients  *vs.*  Alcoholics without ALD | c2 vs. c1 | 6 | 1.06 | 0.63-1.79 | F | 0.822 |  | 45.9 | 0.100 |  | 0.384 |
|  |  | c2c2 vs. c1c1 | 2 | 0.25 | 0.03-2.39 | F | 0.230 |  | 0.0 | 0.687 |  | — |
|  |  | c1c2 vs. c1c1 | 6 | 1.34 | 0.76-2.35 | F | 0.313 |  | 32.0 | 0.196 |  | 0.475 |
|  |  | c2c2+c1c2 vs. c1c1 | 6 | 1.19 | 0.69-2.06 | F | 0.534 |  | 40.7 | 0.134 |  | 0.410 |
|  | Other cases  *vs.*  Alcoholics without ALD | c2 vs. c1 | 4 | 2.58 | 1.42-4.67 | F | 0.002 |  | 2.1 | 0.382 |  | 0.398 |
|  |  | c2c2 vs. c1c1 | 4 | 2.15 | 0.50-9.22 | F | 0.303 |  | 0.0 | 0.840 |  | 0.337 |
|  |  | c1c2 vs. c1c1 | 4 | 2.44 | 1.26-4.73 | F | 0.008 |  | 40.7 | 0.168 |  | 0.951 |
|  |  | c2c2+c1c2 vs. c1c1 | 4 | 2.58 | 1.37-4.87 | F | 0.003 |  | 23.3 | 0.271 |  | 0.718 |
|  | ALC patients  *vs.*  Non-alcoholics | c2 vs. c1 | 9 | 1.24 | 0.62-2.50 | R | 0.546 |  | 63.0 | 0.006 |  | 0.739 |
|  |  | c2c2 vs. c1c1 | 1 | 0.82 | 0.03-20.2 | F | 0.901 |  | — | — |  | — |
|  |  | c1c2 vs. c1c1 | 8 | 1.37 | 0.62-3.01 | R | 0.439 |  | 70.2 | 0.001 |  | 0.789 |
|  |  | c2c2+c1c2 vs. c1c1 | 8 | 1.35 | 0.62-2.95 | R | 0.455 |  | 70.0 | 0.001 |  | 0.805 |
|  | Other cases  *vs.*  Non-alcoholics | c2 vs. c1 | 5 | 2.26 | 0.89-5.70 | R | 0.085 |  | 51.1 | 0.085 |  | 0.850 |
|  |  | c2c2 vs. c1c1 | 3 | 3.60 | 0.60-21.69 | F | 0.163 |  | 0.0 | 0.899 |  | 0.318 |
|  |  | c1c2 vs. c1c1 | 5 | 1.67 | 0.61-4.56 | R | 0.319 |  | 52.8 | 0.076 |  | 0.609 |
|  |  | c2c2+c1c2 vs. c1c1 | 5 | 2.03 | 0.79-5.20 | R | 0.143 |  | 50.2 | 0.090 |  | 0.735 |

^a^ M, model of meta-analysis; F, fixed effect model; R, random effect model.

^b^ *P*_value_, *P* value for heterogeneity based on Q test.

^c^ *P*_Egger’s_ _test_*, P* value for Egger’s test.

“—” Values could not be calculated out.

ALC, alcoholic liver cirrhosis;

Other cases, ALD patient with other type of ALD (such as steatosis, hepatisis, fibrosis, etc) or with a mixture type of ALD.
