# Supplementary material for: Roles of Cytochrome P4502E1 Gene Polymorphisms and the Risks of Alcoholic Liver Disease: A Meta-Analysis
Source: PLoS One. 2013 Jan 15;8(1):e54188. doi: 10.1371/journal.pone.0054188 (PMC3545986; doi:10.1371/journal.pone.0054188)
Supplement: Table S4 — Meta-analysis for the association between CYP2E1 Pst I/Rsa I polymorphism and the risk of ALD in male subjects (DOC) [file pone.0054188.s004.doc]

**Table S4 Meta-analysis for the association between *CYP2E1 Pst I/Rsa I polymor*phism and the risk of ALD in male subjects**

|  |  | Contrasts | No. of studies | Test of association | | | |  | Test of heterogeneity | |
| --- | --- | --- | --- | --- | --- | --- | --- | --- | --- | --- |
|  |  |  |  | OR | 95%CI | M^a^ | *P*_OR_ |  | *I*^2^ (%) | *P*_value_ ^b^ |
| ALD patients  *vs.*  Alcoholics without ALD | All | c2 vs. c1 | 5 | 1.16 | 0.75-1.77 | F | 0.506 |  | 45.8 | 0.117 |
|  |  | c2c2 vs. c1c1 | 4 | 1.80 | 0.55-5.94 | F | 0.334 |  | 32.9 | 0.215 |
|  |  | c1c2 vs. c1c1 | 5 | 1.01 | 0.60-1.68 | F | 0.976 |  | 0.0 | 0.623 |
|  |  | c2c2+c1c2 vs. c1c1 | 5 | 1.08 | 0.66-1.77 | F | 0.751 |  | 15.4 | 0.316 |
|  | Asians | c2 vs. c1 | 2 | 1.59 | 0.63-4.05 | R | 0.329 |  | 67.9 | 0.078 |
|  |  | c2c2 vs. c1c1 | 2 | 3.85 | 0.75-19.94 | F | 0.108 |  | 34.4 | 0.217 |
|  |  | c1c2 vs. c1c1 | 2 | 1.27 | 0.67-2.40 | F | 0.462 |  | 0.0 | 0.386 |
|  |  | c2c2+c1c2 vs. c1c1 | 2 | 1.43 | 0.77-2.63 | F | 0.255 |  | 45.1 | 0.177 |
|  | Caucasians | c2 vs. c1 | 3 | 0.66 | 0.31-1.41 | F | 0.282 |  | 0.0 | 0.525 |
|  |  | c2c2 vs. c1c1 | 2 | 0.59 | 0.08-4.03 | F | 0.587 |  | 23.7 | 0.252 |
|  |  | c1c2 vs. c1c1 | 3 | 0.66 | 0.28-1.53 | F | 0.331 |  | 0.0 | 0.825 |
|  |  | c2c2+c1c2 vs. c1c1 | 3 | 0.65 | 0.29-1.47 | F | 0.301 |  | 0.0 | 0.735 |
| ALD patients  *vs.* Non-alcoholics | All | c2 vs. c1 | 6 | 1.26 | 0.65-2.46 | R | 0.493 |  | 70.2 | 0.005 |
|  |  | c2c2 vs. c1c1 | 4 | 1.99 | 0.21-18.61 | R | 0.548 |  | 52.6 | 0.097 |
|  |  | c1c2 vs. c1c1 | 6 | 1.60 | 0.62-4.17 | R | 0.334 |  | 79.2 | 0.000 |
|  |  | c2c2+c1c2 vs. c1c1 | 6 | 1.64 | 0.62-4.30 | R | 0.318 |  | 80.1 | 0.000 |
|  | Asians | c2 vs. c1 | 3 | 1.69 | 0.66-4.32 | R | 0.270 |  | 82.0 | 0.004 |
|  |  | c2c2 vs. c1c1 | 3 | 2.06 | 0.09-46.46 | R | 0.650 |  | 68.4 | 0.042 |
|  |  | c1c2 vs. c1c1 | 3 | 3.35 | 0.65-17.39 | R | 0.150 |  | 89.1 | 0.000 |
|  |  | c2c2+c1c2 vs. c1c1 | 3 | 3.30 | 0.62-17.7 | R | 0.163 |  | 89.7 | 0.000 |
|  | Caucasians | c2 vs. c1 | 3 | 0.78 | 0.41-1.47 | F | 0.442 |  | 17.7 | 0.297 |
|  |  | c2c2 vs. c1c1 | 1 | 1.91 | 0.08-48.34 | R | 0.695 |  | — | — |
|  |  | c1c2 vs. c1c1 | 3 | 0.70 | 0.36-1.37 | F | 0.299 |  | 0.0 | 0.500 |
|  |  | c2c2+c1c2 vs. c1c1 | 3 | 0.74 | 0.38-1.42 | F | 0.361 |  | 0.0 | 0.397 |

^a^ M, model of meta-analysis; F, fixed effect model; R, random effect model.

^b^ *P*_value_, *P* value for heterogeneity based on Q test.

“—” Values could not be calculated out.

ALD, alcoholic liver diseases.
