# Supplementary material for: Roles of Cytochrome P4502E1 Gene Polymorphisms and the Risks of Alcoholic Liver Disease: A Meta-Analysis
Source: PLoS One. 2013 Jan 15;8(1):e54188. doi: 10.1371/journal.pone.0054188 (PMC3545986; doi:10.1371/journal.pone.0054188)
Supplement: Table S5 — Meta-analysis for the association between CYP2E1 Dra I polymorphism and the risk of ALD (DOC) [file pone.0054188.s005.doc]

**Table S5 Meta-analysis for the association between *CYP2E1 Dra I polymor*phism and the risk of ALD**

|  |  | Contrasts | No. of studies | Test of association | | | |  | Test of heterogeneity | |  | *P*_Egger’s test_ ^c^ |
| --- | --- | --- | --- | --- | --- | --- | --- | --- | --- | --- | --- | --- |
|  |  |  |  | OR | 95%CI | M^a^ | *P*_OR_ |  | *I*^2^ (%) | *P*_value_ ^b^ |  |  |
| cases  *vs.*  alcoholics without ALD | ALD | d1 vs.d2 | 5 | 1.09 | 0.80-1.48 | F | 0.558 |  | 0.0 | 0.795 |  | 0.803 |
|  |  | d1d1 vs.d2d2 | 4 | 0.67 | 0.19-2.43 | F | 0.546 |  | 0.0 | 0.864 |  | 0.592 |
|  |  | d1d2 vs.d2d2 | 5 | 1.14 | 0.81-1.62 | F | 0.447 |  | 0.0 | 0.936 |  | 0.941 |
|  |  | d1d1/d1d2 vs.d2d2 | 6 | 1.00 | 0.75-1.33 | F | 0.975 |  | 0.0 | 0.702 |  | 0.440 |
|  | ALC | d1 vs.d2 | 4 | 1.02 | 0.73-1.42 | F | 0.924 |  | 0.0 | 0.882 |  | 0.707 |
|  |  | d1d1 vs.d2d2 | 3 | 0.56 | 0.13-2.49 | F | 0.444 |  | 0.0 | 0.789 |  | 0.787 |
|  |  | d1d2 vs.d2d2 | 4 | 1.09 | 0.75-1.58 | F | 0.670 |  | 0.0 | 0.949 |  | 0.466 |
|  |  | d1d1/d1d2 vs.d2d2 | 5 | 0.94 | 0.69-1.27 | F | 0.679 |  | 0.0 | 0.783 |  | 0.780 |
| cases  *vs.*  Non-alcoholics | ALD | d1 vs.d2 | 7 | 1.13 | 0.77-1.66 | R | 0.536 |  | 48.0 | 0.073 |  | 0.232 |
|  |  | d1d1 vs.d2d2 | 4 | 0.94 | 0.23-3.81 | F | 0.926 |  | 0.0 | 0.967 |  | 0.484 |
|  |  | d1d2 vs.d2d2 | 6 | 1.20 | 0.73-1.98 | R | 0.477 |  | 59.8 | 0.029 |  | 0.286 |
|  |  | d1d1/d1d2 vs.d2d2 | 7 | 1.13 | 0.76-1.70 | R | 0.542 |  | 56.6 | 0.032 |  | 0.698 |
|  | ALC | d1 vs.d2 | 6 | 1.16 | 0.74-1.86 | R | 0.519 |  | 54.1 | 0.053 |  | 0.260 |
|  |  | d1d1 vs.d2d2 | 3 | 0.88 | 0.18-4.24 | F | 0.872 |  | 0.0 | 0.892 |  | 0.339 |
|  |  | d1d2 vs.d2d2 | 5 | 1.24 | 0.67-2.28 | R | 0.492 |  | 66.0 | 0.019 |  | 0.327 |
|  |  | d1d1/d1d2 vs.d2d2 | 6 | 1.16 | 0.72-1.87 | R | 0.536 |  | 62.9 | 0.019 |  | 0.756 |

^a^ M, model of meta-analysis; F, fixed effect model; R, random effect model;

^b^ *P*_value_, *P* value for heterogeneity based on Q test;

^c^ *P*_Egger’s_ _test_*, P* value for Egger’s test;

“—” Values could not be calculated out;

ALD, alcoholic liver diseases;

ALC, alcoholic liver cirrhosis.
